# A de novo frameshift in HNRNPK causing a Kabuki‐like syndrome with nodular heterotopia

**DOI:** 10.1111/cge.12773

**Published:** 2016-04-01

**Authors:** L. Lange, A. T. Pagnamenta, S. Lise, S. Clasper, H. Stewart, E. S. Akha, G. Quaghebeur, S. J. L. Knight, D. A. Keays, J. C. Taylor, U. Kini

**Affiliations:** ^1^ National Institute for Health Research Biomedical Research Centre, Wellcome Trust Centre for Human GeneticsUniversity of OxfordOxfordUK; ^2^ Oxford Regional Genetics Service, Oxford Radcliffe Hospitals NHS TrustThe Churchill HospitalOxfordUK; ^3^ Department of Clinical GeneticsOxford University Hospitals NHS Foundation TrustOxfordUK; ^4^ Department of NeuroradiologyOxford University Hospitals NHS Foundation TrustOxfordUK; ^5^ Institute of Molecular PathologyViennaAustria

**Keywords:** exome, heterotopia, HNRNPK, Kabuki

## Abstract

Kabuki syndrome is a heterogeneous condition characterized by distinctive facial features, intellectual disability, growth retardation, skeletal abnormalities and a range of organ malformations. Although at least two major causative genes have been identified, these do not explain all cases. Here we describe a patient with a complex Kabuki‐like syndrome that included nodular heterotopia, in whom testing for several single‐gene disorders had proved negative. Exome sequencing uncovered a de novo c.931_932insTT variant in HNRNPK (heterogeneous nuclear ribonucleoprotein K). Although this variant was identified in March 2012, its clinical relevance could only be confirmed following the August 2015 publication of two cases with HNRNPK mutations and an overlapping phenotype that included intellectual disability, distinctive facial dysmorphism and skeletal/connective tissue abnormalities. Whilst we had attempted (unsuccessfully) to identify additional cases through existing collaborators, the two published cases were ‘matched’ using GeneMatcher, a web‐based tool for connecting researchers and clinicians working on identical genes. Our report therefore exemplifies the importance of such online tools in clinical genetics research and the benefits of periodically reviewing cases with variants of unproven significance. Our study also suggests that loss of function variants in HNRNPK should be considered as a molecular basis for patients with Kabuki‐like syndrome.

Over the last decade, the development of NGS technologies has accelerated disease‐gene discovery such that (as of January 2016) there are now reported to be 5654 phenotypes for which the genetic basis is known (www.omim.org/statistics/geneMap). For the remaining rare genetic disorders in which the underlying molecular mechanisms have not yet been determined, online tools such as GeneMatcher 1, GenomeConnect 2 and MyGene2 3 have been developed that can help connect researchers working on the same gene. In this way, rare disease‐causing variants that previously could not be confirmed can now be identified by matching up similar cases from around the world. One such example is a recent study describing two unrelated patients with *de novo* mutations in *HNRNPK,* the gene encoding the heterogeneous nuclear ribonucleoprotein K 4. Both patients presented with intellectual disability, facial dysmorphism and skeletal/connective tissue abnormalities. Structural brain abnormalities such as hypomyelination and thin corpus callosum were also reported. One patient harboured a splice donor site mutation in *HNRNPK* (NM_002140.3:c.953+1dupG) that predicts a frame‐shift and a truncated protein (p.Gly319Argfs*6). In the second patient, a c.257G>A variant, although annotated as p.Arg86His, was shown to be associated with reduced expression of hnRNP K protein, presumably through an aberrant splicing mechanism 4.

Here, we describe a patient (BRC052) with a complex phenotype overlapping that seen in the patients described by Au et al. BRC052 was born following a pregnancy complicated by nuchal thickening of 9.8 mm identified at 12 weeks gestation. Chorionic villus sampling done at the time revealed a normal karyotype. Postnatally, he was found to have an atrioventricular septal defect, cleft palate, renal pelvic dilatation, bilateral talipes and partial agenesis of the corpus callosum. He was hypotonic and showed significant developmental delay. Dysmorphic features included a prominent metopic ridge (Fig. 1a,b), medial flare of the eyebrows, long palpebral fissures with lateral eversion of lower eyelids, hypoplastic alae nasi, low‐set prominent ears and thick, wrinkled skin in the back of the neck, which was already present at birth (Fig. 1c), hypoplastic toe nails and very deep palmar and plantar creases (Fig. 1d,e) and a midline groove on his tongue. He was noted to have a shallow acetabulum and mild scoliosis. CT scan of the skull revealed a saggital synostosis and a repeat MRI brain scan confirmed agenesis of the corpus callosum, with nodular heterotopia in the frontal horns (Fig. 1f). The patient's neonatal weight, length and head circumference were in the 25–50th, 91–98th and on the 50th centile, respectively.

**Figure 1:**
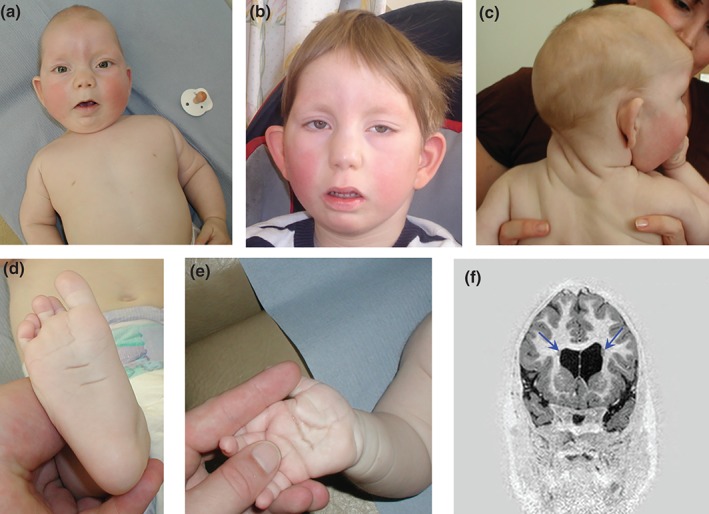
(**a**, **b**) Proband at ages 7 months and 8 years, respectively. Note the prominent metopic ridge, turricephaly, sparseness of lateral eyebrows with medial flare, long palpebral fissures, eversion of lower eyelids, low‐set prominent ears. (**c**) Thick, wrinkled skin on the neck posteriorly. (**d**, **e**) Deep plantar and palmar creases with fetal finger pads. (**f**) Coronal MRI image from inversion recovery volume sequence demonstrating two grey matter rounded nodules (blue arrows) adjacent to the borders of the lateral ventricles, in keeping with periventricular heterotopia. Written consent from the family to publish these images was obtained.

His growth continued on this trajectory such that when last examined at the age of 11 years, his weight, height and head circumference were on the 25th, 91st and in the 25–50th centile range, respectively. He was able to crawl, bottom‐shuffle and walk with help. He had taken 25 steps independently. He had a single word in his speech (‘mum’), although he did make some vowel sounds and was able to communicate with more than 100 signs. His understanding of speech was at a 3‐ to 4‐year old level. He was in the process of being toilet‐trained and attended a school for children with special needs. He had a high threshold for pain.

Testing for several single gene disorders was carried out including Smith Lemli‐Opitz syndrome, Goldberg Shprintzen syndrome, *FLNA*‐associated periventricular heterotopia and Kabuki syndrome (*KMT2D* and *KDM6A*). He was recruited into the Genetics of Structural Brain Abnormalities and Learning Disabilities Study. CNV analysis using 180k array‐CGH (Agilent) identified a rare maternally inherited 82 kb deletion involving *IQGAP2* of uncertain significance but no other likely pathogenic alterations. Whole‐exome sequencing (WES) was performed on the patient and his biological parents using a pipeline that involved SureSelect capture (Agilent), sequencing using the HiSeq2000 (Illumina) and a 100 bp paired‐end read format. The bioinformatic analysis was similar to that described previously 5 using population allele frequencies from ExAC V0.3 (http://exac.broadinstitute.org/) and 1000 Genomes Project (1000G) datasets 6. None of the 415 rare (<1% allele frequency), predicted deleterious variants detected in the patient resided in known Kabuki disease genes (*KMT2D*, *KDM6A*, *KDM1A*) or lay *in trans* with the *IQGAP2* deletion. As BRC052 had no family history of the disease, we scanned for *de novo* variants and detected a single such variant, c.931_932insTT in *HNRNPK*. The variant has not been reported in population‐based cohorts including the ExAC browser which included WES data from 60,706 unrelated individuals and was not observed in 1000G. This mutation is predicted to result in a frameshift (p.Pro311Leufs*40) 67% through the coding sequence, in a region engaging with Zik1, a transcriptional repressor that interacts with hnRNP K 7 and before the third KH domain of hnRNP K (Fig. 2a), which is involved in RNA or ssDNA binding 8. The mutation lies in exon 11 which is not subject to alternative splicing and thus is likely to constitute a LoF allele, similar to those reported by Au et al.

**Figure 2:**
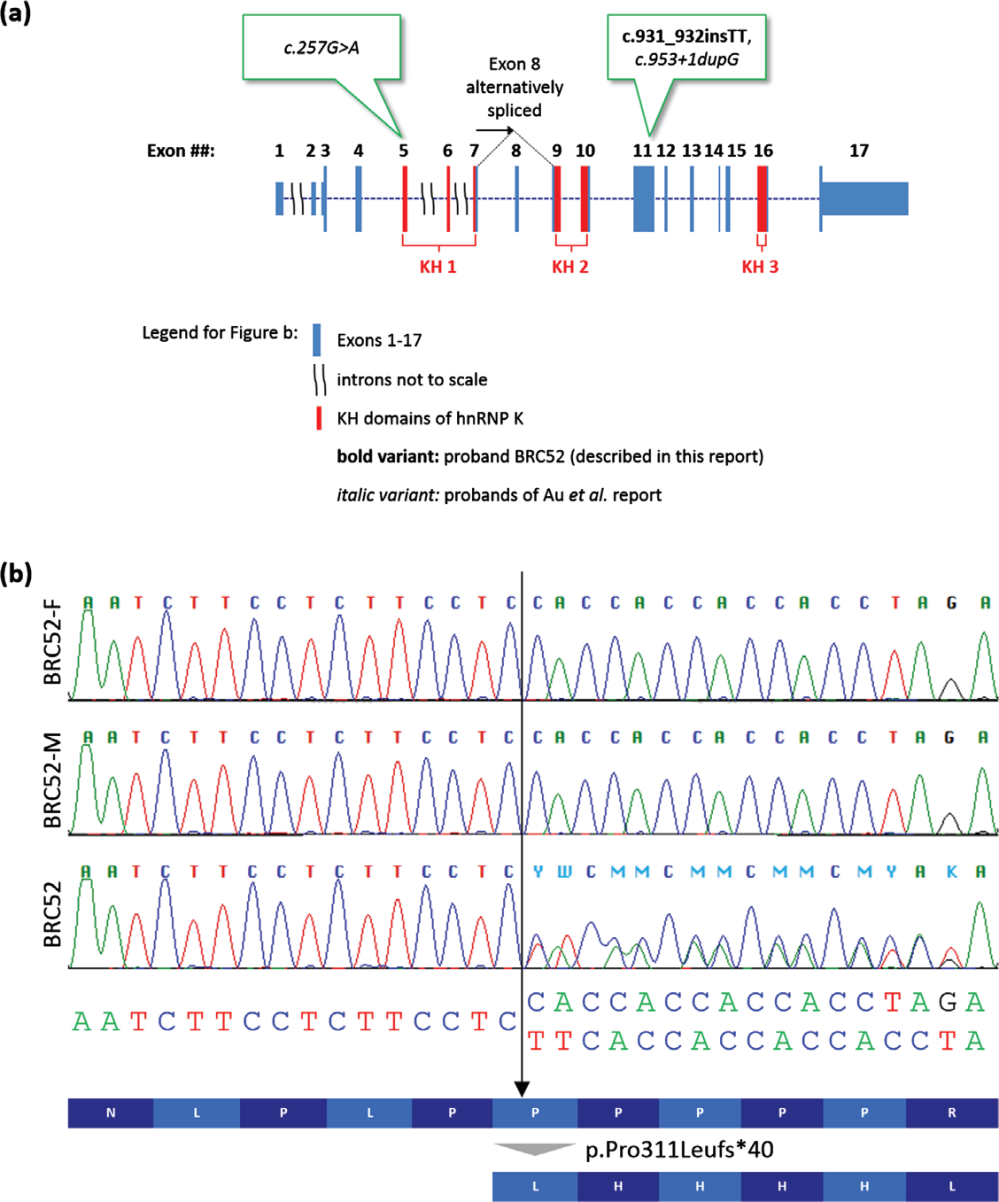
(**a**) Gene structure with position of variants in relation to known functional domains. (**b**) Sanger sequencing electropherogram showing validation results for the c.931_932insTT variant alongside the predicted effect at the amino‐acid level. Sequencing was also performed in the non‐coding direction (data not shown).

At the time of discovery, tools such as GeneMatcher and GenomeConnect were not available and although our data were shared with multiple international collaborators, the c.931_932insTT variant could not be confirmed as being pathogenic. Nevertheless, as it was (i) *de novo*, (ii) a LoF allele, and (iii) in a gene with important roles in chromatin remodelling, transcription, signal transduction and splicing 9 as well as synaptic transmission and plasticity in hippocampal neurons 10, 11, it was considered to be a strong candidate. Therefore, Sanger validation was performed in March 2012. Three years later, following the Au et al. publication in July 2015, and re‐review of exome data, the c.931_932insTT variant (ClinVar accession SCV000258957) was validated in a clinical genetics laboratory (Fig. 2b) and was reported back to the family in December 2015 (Fig. 3). This information was very helpful for the family as they finally have a diagnosis for their child and have been reassured about their own reproductive risks and that of other family members. Prenatal testing will also be on offer to the parents in a future pregnancy due to a small theoretical risk of gonadal mosaicism. This study thus exemplifies the clinical importance of periodically re‐reviewing WES and Whole Genome Sequencing (WGS) data and lists of variants of suspected but unproven significance. This may be particularly challenging for clinical diagnostic labs with limited resources. Large‐scale studies such as the UK's 100,000 Genomes Project would benefit from integrating such review into their pipeline.

**Figure 3:**
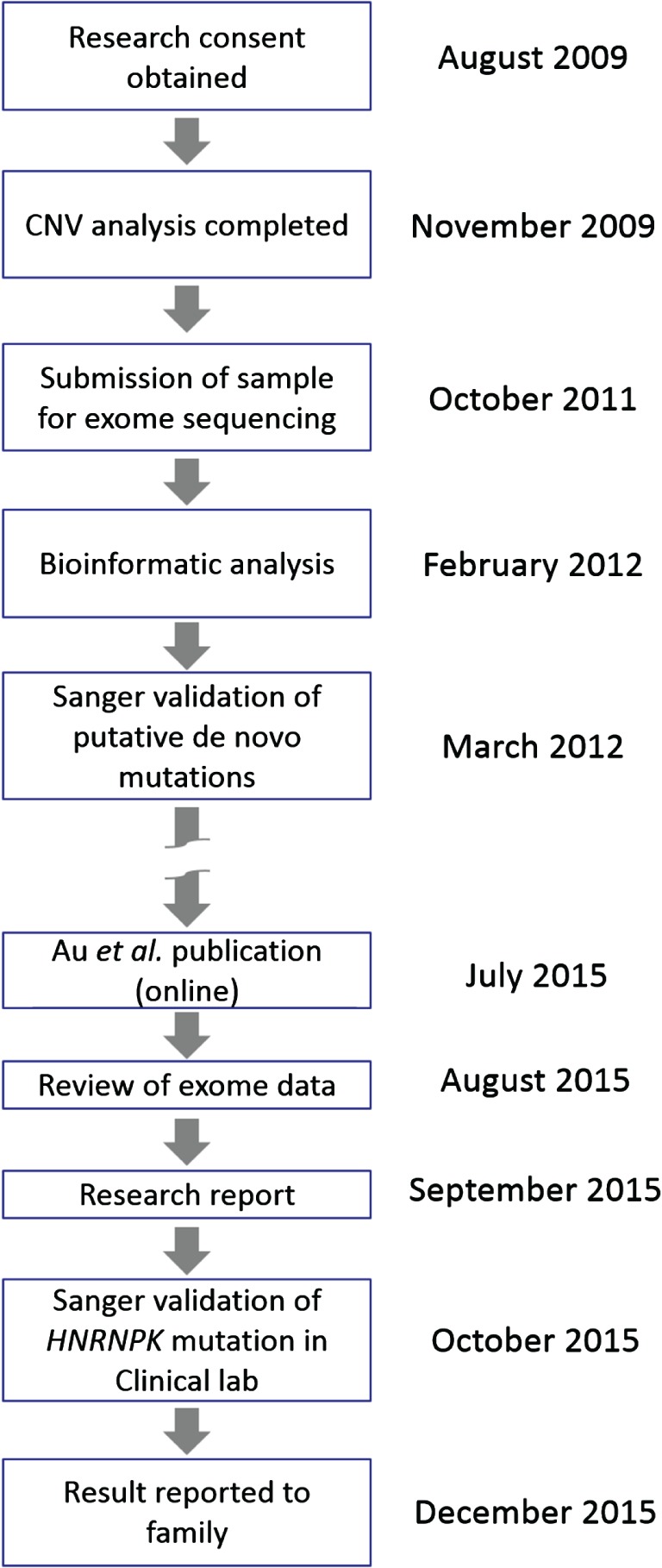
Timeline of significant events leading to the mutation being reported back to the family.

Together with a recent analysis of mutational constraint in the >60k ExAC exomes which shows this gene has a high probability of intolerance to LoF variants (expected number of LoF mutations = 23.8, observed number = 0; pLI = 1.00; Lek *et al*, *in preparation*), our data strengthen the link between *HNRNPK* haploinsufficiency and the complex syndromic phenotype described. *HNRNPK* has an important role in chromatin remodelling and all genes known to cause Kabuki syndrome are chromatin regulators. This might explain the phenotypic overlap seen between Kabuki syndrome and *HNRNPK* mutations. Indeed, Kabuki syndrome was also considered as a potential diagnosis for one of the original cases until *KMT2D* had been excluded 4. A frequent early, prenatal finding in individuals with *HNRNPK* mutations is the increased nuchal thickness. We therefore propose that *HNRNPK* mutations should be kept in mind in case of antenatal scan findings of raised nuchal thickness with cardiac and/or renal anomalies. Craniosynostosis, particularly involving the saggital sutures, also appears to be a frequent finding. A variety of structural brain abnormalities may occur including nodular heterotopia. Facial dysmorphism and multiple congenital anomalies (cardiac, renal and orofacial clefting) overlap with Kabuki syndrome and hence LoF variants in *HNRNPK* should be considered as a molecular basis for patients with Kabuki‐like syndrome.
